# Zinc-Dependent Protection of Tobacco and Rice Cells From Aluminum-Induced Superoxide-Mediated Cytotoxicity

**DOI:** 10.3389/fpls.2015.01079

**Published:** 2015-12-01

**Authors:** Cun Lin, Ayaka Hara, Diego Comparini, François Bouteau, Tomonori Kawano

**Affiliations:** ^1^Faculty of Environmental Engineering and Graduate School of Environmental Engineering, The University of Kitakyushu, Kitakyushu, Japan; ^2^International Photosynthesis Industrialization Research Center, The University of Kitakyushu, Kitakyushu, Japan; ^3^University of Florence LINV Kitakyushu Research Center, Kitakyushu, Japan; ^4^International Plant Neurobiology Laboratory, University of Florence, Sesto Fiorentino, Italy; ^5^Institut des Energies de Demain (FRE3597), Université Paris Diderot, Sorbonne Paris Cité, Paris, France; ^6^Université Paris Diderot, Sorbonne Paris Cité, Paris 7 Interdisciplinary Energy Research Institute, Paris, France

**Keywords:** aluminum, zinc, BY-2, *Nicotiana tabacum* L., *Oryza sativa* L., ROS

## Abstract

Al^3+^ toxicity in growing plants is considered as one of the major factors limiting the production of crops on acidic soils worldwide. In the last 15 years, it has been proposed that Al^3+^ toxicity are mediated with distortion of the cellular signaling mechanisms such as calcium signaling pathways, and production of cytotoxic reactive oxygen species (ROS) causing oxidative damages. On the other hand, zinc is normally present in plants at high concentrations and its deficiency is one of the most widespread micronutrient deficiencies in plants. Earlier studies suggested that lack of zinc often results in ROS-mediated oxidative damage to plant cells. Previously, inhibitory action of Zn^2+^ against lanthanide-induced superoxide generation in tobacco cells have been reported, suggesting that Zn^2+^ interferes with the cation-induced ROS production via stimulation of NADPH oxidase. In the present study, the effect of Zn^2+^ on Al^3+^-induced superoxide generation in the cell suspension cultures of tobacco (*Nicotiana tabacum* L., cell-line, BY-2) and rice (*Oryza sativa* L., cv. Nipponbare), was examined. The Zn^2+^-dependent inhibition of the Al^3+^-induced oxidative burst was observed in both model cells selected from the monocots and dicots (rice and tobacco), suggesting that this phenomenon (Al^3+^/Zn^2+^ interaction) can be preserved in higher plants. Subsequently induced cell death in tobacco cells was analyzed by lethal cell staining with Evans blue. Obtained results indicated that presence of Zn^2+^ at physiological concentrations can protect the cells by preventing the Al^3+^-induced superoxide generation and cell death. Furthermore, the regulation of the Ca^2+^ signaling, i.e., change in the cytosolic Ca^2+^ ion concentration, and the cross-talks among the elements which participate in the pathway were further explored.

## Introduction

Aluminum is the most abundant metal and the third most abundant chemical element in the Earth’s crust. The increase in free aluminum ions (chiefly, Al^3+^) accompanying soil acidification is considered to be toxic to plants (Poschenrieder et al., 2008) and animals (Markich et al., 2002). Al^3+^ toxicity in growing plants is considered as one of the major factors limiting the production of crops on acidic soils worldwide (Poschenrieder et al., 2008; Panda et al., 2009).

A number of studies documented the toxic impact of Al^3+^ especially on roots (Le Van et al., 1994; Lukaszewski and Blevins, 1996; Sanzonowicz et al., 1998). It has been proposed that early effects of Al^3+^ toxicity at growing root apex, such as those on cell division, cell extension or nutrient transport, involve the binding to (Ma et al., 1999) or uptake of Al^3+^ by plants (Lazof et al., 1994; Babourina and Rengel, 2009). Accordingly, actin cytoskeleton and vesicle trafficking are primary targets for Al^3+^ toxicity in the root tips of the sensitive variety (Amenós et al., 2009).

In the last 15 years, it has been proposed that Al^3+^ toxicity are mediated with distortion of the cellular signaling mechanisms such as calcium signaling pathways (Kawano et al., 2003a, 2004; Rengel and Zhang, 2003; Lin et al., 2005, 2006a), and production of cytotoxic reactive oxygen species (ROS) causing oxidative damages (Yamamoto et al., 2002; Kawano et al., 2003a). Recently, Al^3+^-induced DNA damages in the root cells of *Allium cepa* was shown to be blocked by calcium channel blockers suggesting that Al^3+^-stimulated influx of extracellular Ca^2+^ into cytosol causes the programmed cell death-like decomposition of DNA (Achary et al., 2013).

To date, two independent groups have proposed the likely modes of ROS production in Al^3+^-treated plant cells. While Yamamoto et al. (2002), propounded the role of mitochondria challenged by Al^3+^ using the cultured cells of tobacco (*Nicotiana tabacum* L., cell line SL) and the roots of pea (*Pisum sativum* L.); our group (Kawano et al., 2003a) emphasized the involvement of NADPH oxidase, thus sensitive to an inhibitor of NADPH oxidase, diphenylene iodonium (DPI) in tobacco BY-2 cells. While ROS is slowly produced through mitochondrial dysfunction (*ca*. 12 h after Al^3+^ treatment; Yamamoto et al., 2002), the NADPH oxidase-mediated production of superoxide anion radical (O_2_^•–^) takes place immediately after Al^3+^ treatment (Kawano et al., 2003a).

The action of Al^3+^ for induction of O_2_^•–^ generation which is sensitive to DPI was recently confirmed in the cells of *Arabidopsis thaliana* (Kunihiro et al., 2011). Furthermore, the Al^3+^-induced oxidative burst showed biphasic signature consisted with an acute transient spike and a slow but long-lasting wave of O_2_^•–^ generation. In addition, among six respiratory burst oxidase homologs (*Atrbohs*) coding for plant NADPH oxidase, solely *AtrbohD* was shown to be responsive to Al^3+^ in biphasic manner by showing rapid (1 min) and long-lasting (24 h) expression profiles (Kunihiro et al., 2011).

Interestingly, the mechanism of Al^3+^-induced oxidative burst (production of O_2_^•–^) is highly analogous to the response of tobacco cell suspension culture to other metal cations, chiefly trivalent cations of lanthanides such as La^3+^ and Gd^3+^ (Kawano et al., 2001). Therefore, we assume that some known chemical factors reportedly interfere with the lanthanide-induced plant oxidative burst might be active for protection of plant cells from Al^3+^-induced oxidative stress. Such chemicals of interest to be tested include zinc and manganese (Kawano et al., 2002).

Zinc is normally present in plants at high concentrations (Santa-Maria and Cogliatti, 1988) and its deficiency is one of the most widespread micronutrient deficiencies in plants, causing severe reductions in crop production (Cakmak, 2000). Increasing studies indicate that oxidative damage to cellular components caused in plants being challenged by ROS, is highly due to the deficiency of zinc (Pinton et al., 1994; Cakmak, 2000).

Previously, inhibitory action of Zn^2+^ against lanthanide-induced O_2_^•–^ generation in tobacco cells have been reported (Kawano et al., 2002). Pretreatments with Zn^2+^ reportedly interferes the La^3+^- and Gd^3+^-induced O_2_^•–^ generation in tobacco cells. In the tobacco model, Zn^2+^ was shown to minimize the earlier phase of lanthanide-induced O_2_^•–^ production while allowing the release of O_2_^•–^ in the later phase, thus causing the retardation of the lanthanide actions on O_2_^•–^ generation.

Although this process is well known, if it is preserved in higher plants and the specific mechanism of action have is not still clear. For this reason, in the present study, effect of Zn^2+^ on Al^3+^-induced O_2_^•–^ generation in the suspension cultures of tobacco BY-2 cells and rice (*Oryza sativa* L., cv. Nipponbare) cells, was examined. Furthermore, the regulation of the Ca^2+^ signaling, i.e., change in the cytosolic Ca^2+^ ion concentration ([Ca^2+^]_*c*_), and the cross-talks among the elements which participate in the pathway were further explored. Finally the possible use of Zn^2+^ for protection of plant cells from Al^3+^ toxicity is discussed.

## Materials and Methods

### Chemicals

O_2_^•–^-specific chemiluminescence (CL) probe, *Cypridina* luciferin analog (CLA; 2-methyl-6-phenyl-3,7-dihydroimidazo[1,2-a]pyrazin-3-one) designated as CLA was purchased from Tokyo Kasei Kogyo Co. (Tokyo, Japan). Aluminum (III) chloride hexahydrate (AlCl_3_·6H_2_0), zinc sulfate heptahydrate (ZnSO_4_·7H_2_O), gadolinium chloride hexahydrate (GdCl_3_·6H_2_O), and salicylic acid (SA) were from Wako Pure Chemical Industries (Osaka, Japan). Lanthanum chloride heptahydrate (LaCl_3_·7H_2_O) was from Kanto Chemical Co., Inc (Tokyo, Japan). DPI chloride, Evans blue, 4,5-dihydroxy-1,3-benzene-disulfonic acid (Tiron), *N*,*N*^′^-dimethylthiourea (DMTU), were from Sigma (St. Louis, MO, USA). Coelenterazine was a gift from Prof. M. Isobe (Nagoya University).

### Plant Cell Culture

**FIGURE 1:**
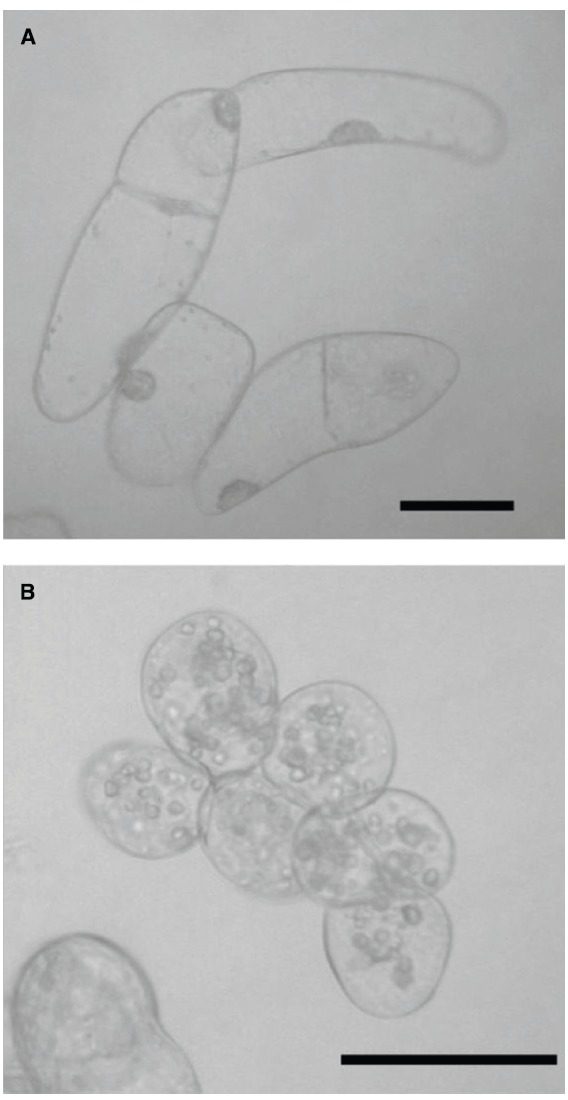
**Microscopic images of plant cells used in this study. (A)** Tobacco BY-2 cells. **(B)** Rice M1 cells. Scale bars, 50 μm.

Tobacco (*Nicotiana tabacum* L. cv. Bright Yellow-2) suspension-culture cells (cell line, BY-2, expressing the aequorin gene; Figure 1A) were propagated as previously described (Kawano et al., 1998). Briefly, the culture was maintained in Murashige–Skoog liquid medium (pH 5.8) supplemented with 3% (w/v) sucrose and 0.2 μg ml^–1^ of 2,4-dichlorophenoxyacetic acid. The culture was propagated with shaking on a gyratory shaker in darkness at 23°C. For sub-culturing, 1.0 ml of confluent stationary culture was suspended in 30 ml of fresh culture medium and incubated at 23°C with shaking at 130 rpm on a gyratory shaker in darkness until used.

Rice callus tissues (*Oryza sativa* L., cv. Nipponbare, cell line, M1; Figure 1B) were obtained from root explants derived from young seedlings and transferred in AA liquid medium to develop a suspension-culture. The cells were maintained and propagated at 23°C with shaking at 130 rpm on a gyratory shaker in darkness. For sub-culturing, with 2-week intervals, 10 ml of stationary culture was suspended in 100 ml of fresh culture medium.

### Detection of O_2_^•–^ with CLA

To detect the production of O_2_^•–^ in plant cells, the 200 μl of plant cell suspension (either of tobacco or rice) was placed in glass cuvettes and CLA was added at final concentration of 2 μM (in tobacco cells) and 4 μM (in rice cells). The glass cuvettes containing 200 μl of plant cell suspension were placed in luminometers (CHEM-GLOW Photometer, American Instrument Co., Silver Spring, MD, USA; or PSN AB-2200-R Luminescensor, Atto, Tokyo). Generation of O_2_^•–^ in cell suspension culture was monitored by CLA-CL, and expressed as relative chemiluminescence units (rcu) as previously described (Kawano et al., 1998). CLA-CL specifically indicates the generation of O_2_^•–^ (and of ^1^O_2_ with a minor extent) but not that of O_3_, H_2_O_2_ or hydroxy radicals (Nakano et al., 1986).

### Aequorin Ca^2+^ Detection

To detect the changes in [Ca^2+^]_*c*_ in tobacco cells, 10 mL of plant cell suspension were pre-treated for 8 h with 10 μL of coelenterazine in the dark, then used for the experiments as previously described (Kawano et al., 1998). Also in this case, 200 μl plant of cell suspension was placed in glass cuvettes and placed in luminometers (as above). Increase in [Ca^2+^]_*c,*_ reflecting the induced Ca^2+^ into cells, was monitored as luminescence derived upon binding of Ca^2+^ to aequorin (the recombinant gene over-expressed in the cytosol) and expressed as rcu.

### Treatments with Aluminum, Zinc, and Other Stimuli

Tobacco BY-2 cells were harvested various days after sub-culturing (as indicated), and used for experiments with CLA or aequorin. AlCl_3_ was dissolved in distilled water and diluted with fresh culture media unless indicated, and 10–20 μl of the AlCl_3_ solution was added to 180–190 μl of cell suspension in glass cuvettes, and level of [Ca^2+^]_*c*_ (aequorin experiment) or generation of O_2_^•–^ (CLA experiment) were monitored. For comparison, effects of SA and hypo-osmotic shock (induced by dilution of media giving Δ100 mOsmol of hypo-osmolarity difference) on calcium homeostasis with and without zinc was monitored. Inhibition of events induced by Al^3+^ and other stimuli, monitored with CLA CL, aequorin luminescence, and cell death staining, was performed by addition of indicated concentration of Zn^2+^ to the cells prior to treatments with Al^3+^, SA, and hypo-osmotic shock.

### Monitoring of Cell Death

Al^3+^-induced cell death in BY-2 tobacco cell suspension culture was allowed to develop in the presence of Evans blue, a lethal staining dye (0.1%, w/v). Evans blue was added to the cell suspension culture, 6 h after Al^3+^ application unless indicated or at the time indicated (0–8 h after Al addition). Then, the cells were further incubated for 1 h for fully developing and detecting the cell death as described (Kadono et al., 2006). After terminating the staining process by washing, stained cells were counted under microscopes. For statistical analyses, four different digital images of cells under the microscope (each covering 50 cells to be counted) were acquired and stained cells were counted.

## Results and Discussions

### Induction of O_2_^•–^

**FIGURE 2:**
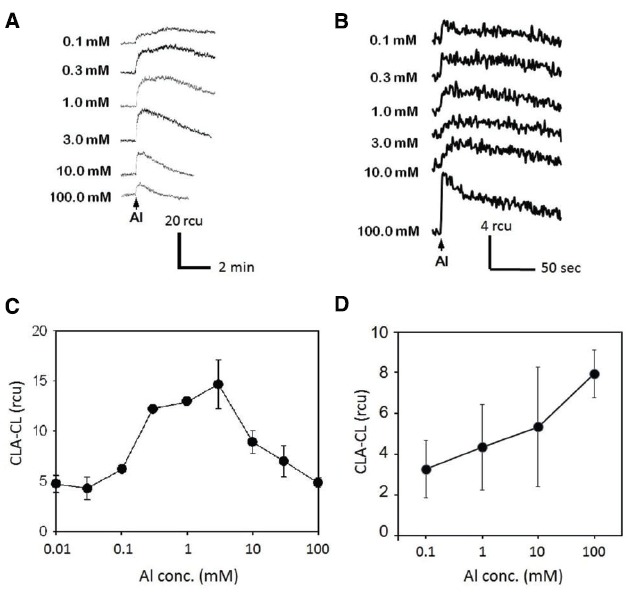
**Effect of Al^3+^ concentration on O_2_^•–^ induction.** Typical records of Al^3+^-induced O_2_^•–^ measured with CLA-CL in the cell suspension cultures of tobacco **(A)** and rice **(B)**. Effect of Al^3+^ concentration on O_2_^•–^ generation (**C**, tobacco; **D**, rice). Vertical error bars, SD; *n* = 3.

The effect of Al^3+^ concentration on induction of O_2_^•–^ generation has been tested both in tobacco and rice cell suspension cultures (Figure 2). For this analysis, BY-2 tobacco cells have been tested 4 days after inoculation (DAI) unless indicated whereas the rice cell line M1 suspension culture was used 14 DAI, since the tobacco BY-2 cells grow at faster rate compared to rice M1 cells. In tobacco BY-2 cells, the active Al^3+^ concentrations for induction of O_2_^•–^ generation ranged from 0.1 mM to 30 mM (optimally at 3 mM).

Notably, higher concentration of Al^3+^ was shown to be inhibitory to induction of O_2_^•–^ generation in the tobacco cells (Figures 2A,C), while the rice cells showed only the proportional increase in generation of O_2_^•–^ with the increase in Al^3+^ up to 100 mM (Figures 2B,D). In order to analyze the impact of Zn^2+^ against Al^3+^-induced O_2_^•–^ generation, the concentration of Al^3+^ was fixed to at 3 mM for the tobacco cells and 100 mM for rice cells. Different concentrations have been chosen since the tobacco cells showed higher sensitivity to relatively lower concentrations of Al^3+^.

### Effect of Pretreatment with Zn^2+^

**FIGURE 3:**
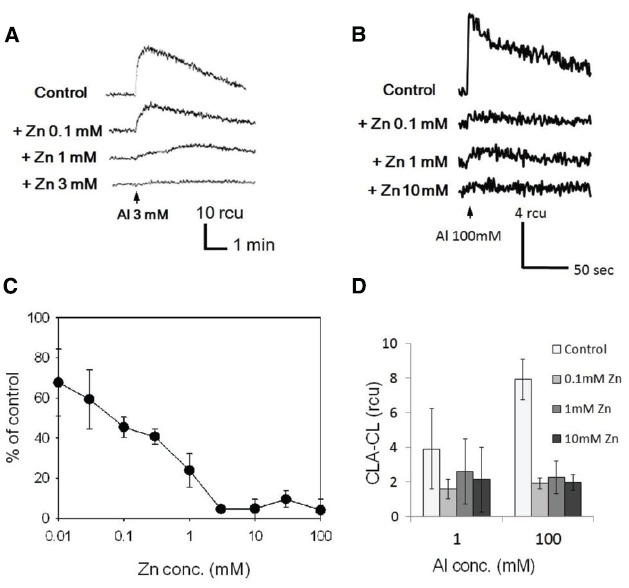
**Inhibition of the Al^3+^-induced generation of O_2_^•–^ in the presence of Zn^2+^.** Typical records of Al^3+^-induced O_2_^•–^ generation in the absence and the presence of ZnSO_4_ measured with CLA (**A**, tobacco; **B**, rice). Arrows indicate the time points for addition of AlCl_3_ (3 mM, tobacco; 100 mM, rice). Effects of Zn^2+^ concentration on Al^3+^-induced O_2_^•–^ generation (**C**, tobacco; **D**, rice). Vertical error bars, SD; *n* = 3.

To assess the effect of Zn^2+^, the cells of tobacco and rice were pre-treated with various concentration of ZnSO_4_ for 5 min and then AlCl_3_ was added to the cells (Figure 3). In tobacco cell, the O_2_^•–^ generation induced by 3 mM Al^3+^ was significantly inhibited by 1 mM or higher concentrations of Zn^2+^, whilst in rice cell, 0.1 mM of Zn^2+^ was high enough to achieve a significant inhibition of O_2_^•–^ generation induced by 100 mM Al^3+^. Although Zn^2+^-dependent retardation of lanthanide-induced O_2_^•–^ production has been reported (Kawano et al., 2002), the Al^3+^-induced oxidative burst was simply inhibited without allowing the onset of slower increase in O_2_^•–^ production.

The Zn^2+^-dependent inhibition was observed in both model cells selected from the monocots and dicots (rice and tobacco), suggesting that this phenomenon (Al^3+^/Zn^2+^ interaction) can be observed universally in the wide range of higher plants. Since the sensitivity was higher in tobacco BY-2 cells, this cell line was chosen to be used in the further experiments examining the mode of Al^3+^/Zn^2+^ interaction.

### Effect of Culture Age on O_2_^•–^ Production

**FIGURE 4:**
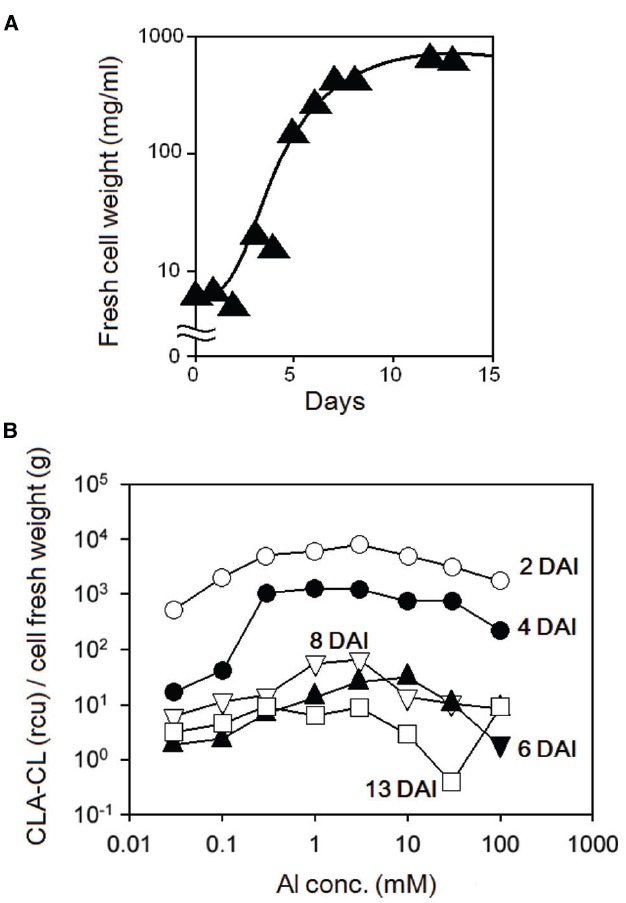
**Effect of culture age on Al^3+^-induced O_2_^•–^ generation in tobacco cell suspension culture. (A)** Growth curve for tobacco cells. **(B)** Four different patterns of CLA-CL reflecting the Al^3+^-induced O_2_^•–^ generation.

Prior to treatment with Al^3+^, tobacco BY-2 cell suspension culture was aged for 1, 3, 5, 8, and 13 DAI of the fresh media (30 ml) with 0.5 ml of confluent culture (at 10 DAI). The cultures at 1 and 3 DAI were smooth and colorless. The 4 and 5 DAI cultures were also smooth but colored slightly yellowish. The 8 and 12 DAI cultures were highly dense and colored yellow. The growth of the culture was assessed by measuring the changes in fresh cell weight at each time point. Figure 4A shows a typical growth curve for tobacco BY-2 cell culture. Effect of culture age of tobacco BY-2 cells on the sensitivity to Al^3+^ was examined using the differently aged cultures (Figure 4B), and the high sensitivity to Al^3+^ was observed in 2 and 4 DAI of tobacco BY-2 cells.

### Competition Between Zn^2+^ and Al^3+^

**FIGURE 5:**
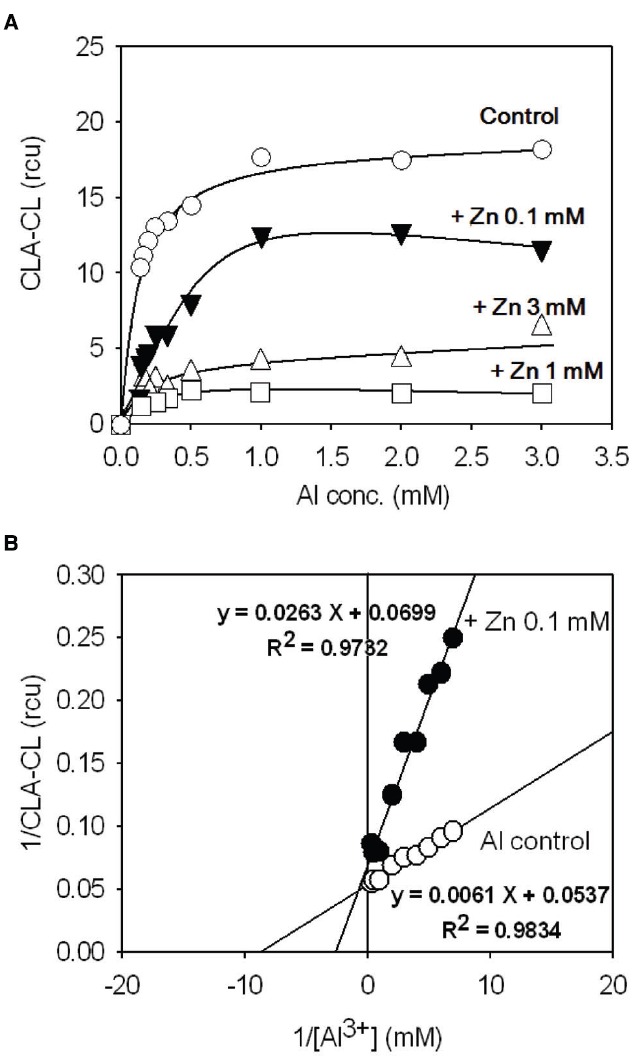
**Competitive inhibition of the Al^3+^-induced O_2_^•–^ generation by Zn^2+^ in tobacco cell suspension culture. (A)** Effect of Zn^2+^ on Al^3+^-induced O_2_^•–^ generation. **(B)** *In vivo* Lineweaver–Burk plot analysis.

Application of double-reciprocal plot analysis for studying the behavior of living plants or cells, so-called *in vivo* Lineweaver–Burk plot analysis was carried out to assess the mode of Al^3+^/Zn^2+^ interaction according to the procedure described elsewhere (Kawano et al., 2003b). By making use of linear dose-dependency in the limited range of Al^3+^ concentrations (up to 3 mM) in 4 DAI culture of tobacco BY-2 cell, the *in vivo* kinetic analysis was carried out by assuming Al^3+^ as a ligand to the putative Al^3+^ receptors on the cells and Zn^2+^ as an inhibitor (Figure 5A). The reciprocals of the CLA-CL yields (1/CLA-CL) were plotted against the reciprocals of Al^3+^ concentrations (1/[Al^3+^]). Linear relationship between 1/CLA-CL and 1/[Al^3+^] were obtained both in the presence and absence of Zn^2+^ (Figure 5B). In the presence of Zn^2+^, the apparent K_*m*_ for Al^3+^ was elevated from 113 μM (control) to 376 μM (0.1 mM Zn^2+^; *ca*. 3.3-fold increase), while V_*max*_ for Al^3+^-induced CLA-CL was not drastically altered. V_*max*_ for Al^3+^-induced response in the absence of Zn^2+^ was calculated to be 14.3 rcu. In the presence of 0.1 mM Zn^2+^, V_*max*_ was 18.6 rcu (*ca*. 30% increase). Therefore, the mode of Zn^2+^ action against Al^3+^ can be considered as a typical competitive inhibition.

According to Kawano et al. (2003a) the Al^3+^-induced generation of O_2_^•–^ in tobacco cells is catalyzed by Al^3+^-stimulated NADPH oxidase which is sensitive to DPI. The cation-dependent enhancement in NADPH oxidase-catalyzed O_2_^•–^ production is also known in human neutrophils in which binding of metal cations possibly results in spontaneous activation of the O_2_^•–^-generating activity of the membrane-bound enzyme (Cross et al., 1999). We can assume that NADPH oxidase itself, localized on the surface of cells (or other factors associated with NADPH oxidase), behaves as the receptor for Al^3+^ ions. The competitive mode of Zn^2+^ action against the Al^3+^-induced oxidative burst suggests us to consider that the binding site for Al^3+^ and Zn^2+^ on the NADPH oxidase or on the factors associated nearby must be identical.

### Al^3+^-Induced Cell Death and its Inhibition by Zn^2+^

**FIGURE 6:**
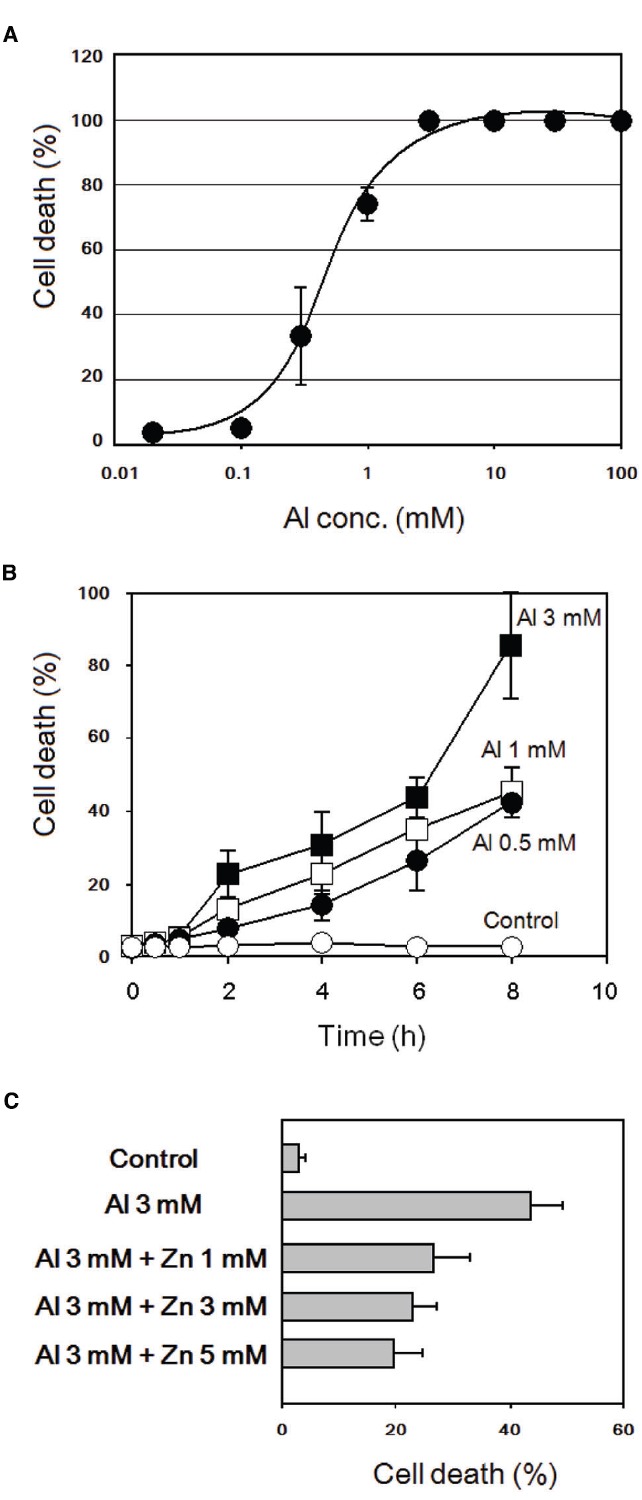
**Al^3+^-induced cell death and its inhibition by addition of Zn^2+^ in tobacco BY-2 cells. (A)** Effect of AlCl_3_ concentration on cell death induction. Cell death was assessed by Evans blue staining 8 h after addition of AlCl_3_. **(B)** Effect of post-Al^3+^ incubation (0.5–8 h) on development of cell death. **(C)** Effect of ZnSO_4_ on Al^3+^-induced cell death. Cell death was assessed 6 h after Al^3+^ treatments. Vertical error bars, SD; *n* = 3.

As shown in Figures 6A,B, treatment of tobacco BY-2 cells with various concentrations of AlCl_3_ resulted in cell death induction. Notably, the presence of Zn^2+^ significantly protected the cells from the induction of cell death by Al^3+^ (Figure 6C), as predicted by the action of Zn^2+^ against Al^3+^-induced oxidative burst.

### Effect of Pretreatment with Mn^2+^

Manganese is another micronutrient possibly protecting the living cells from oxidative damage (Ledig et al., 1991) and reportedly blocks the lanthanide-induced oxidative burst (Kawano et al., 2002). In fact, Mn^2+^ is often employed as a scavenger of O_2_^•–^ for preventing the biochemical reactions involving O_2_^•–^ (Momohara et al., 1990).

Therefore, we tested the effect of MnSO_4_ (up to 3 mM) for comparison. The results obtained suggested no inhibitory effect of Mn^2+^ against Al^3+^-induced generation of O_2_^•–^. Instead, low concentrations of Mn^2+^ slightly elevated the level of Al^3+^-induced oxidative burst (data not shown). For inhibition of Al^3+^-induced oxidative burst, much higher concentrations of MnSO_4_ (10–100 mM) were required. Since the range of Mn^2+^ concentrations required for lowering the level of Al^3+^-induced generation of O_2_^•–^ was at phytotoxic range (Caldwell, 1989) and thus inducing cell death even in the absence of Al^3+^ in BY-2 cells (*ca*. 40% of cells died in the presence of 30 mM MnSO_4_), the use of Mn^2+^ is not suitable for preventing the production of O_2_^•–^ induced by Al^3+^.

### Anti-Oxidative Role for Zn^2+^

Plants require trace amounts of specific metals known as trace nutrients including Zn^2+^, supporting the essential functions of plant cells ranging from respiration to photosynthesis, and molecular biological studies on the mechanism for uptake of these metals by plants have been documented (Delhaize, 1996). One of the important roles for Zn^2+^ in living plants is anti-oxidative action against ROS (Kawano et al., 2002) as the present study successfully demonstrated that extracellular supplementation of Zn^2+^ inhibits the generation of O_2_^•–^ (Figure 4) and cell death (Figure 6C) induced by Al^3+^.

In contrast to manganese, zinc is normally present in plants at high concentrations. For example, in roots of wheat seedlings, the cytoplasmic concentration of total Zn has been estimated to be approximately 0.4 mM (Santa-Maria and Cogliatti, 1988), and Zn-deficiency often results in inhibition of growth, as Zn reportedly protects the plants by preventing the oxidative damages to DNA, membranes, phospholipids, chlorophylls, proteins, SH-containing enzymes, and indole-3-acetic acid (Cakmak, 2000).

Here, Zn^2+^ at sub-mM concentrations showed strong inhibitory action against the toxicity of Al^3+^ (oxidative burst and cell death). The levels of Zn^2+^ naturally present in soil or plant tissues may be contributing to the prevention of Al^3+^-induced cellular damages but further studies on living plants are needed to evaluate this mechanism in living tissue and the possible applications to increase plant tolerance.

### Oxidative and Calcium Crosstalk

Al^3+^ is known to inhibit plant calcium channels similarly to the action of various lanthanide ions (Lin et al., 2006b). The calcium channels sensitive to Al^3+^ could be identical to those involved in responses to ROS (Kawano et al., 2003a, 2004), cold shock (Lin et al., 2005, 2006a, 2007), and heat shock (Lin et al., 2007), but not responsive to osmotic shock (Lin et al., 2005, 2006b, 2007), as examined in transgenic cell lines of rice (*Oryza sativa* L., cv. Nipponbare) and tobacco (cell-lines, BY-2, Bel-B, and Bel-W3) all expressing aequorin in the cytosolic space.

**FIGURE 7:**
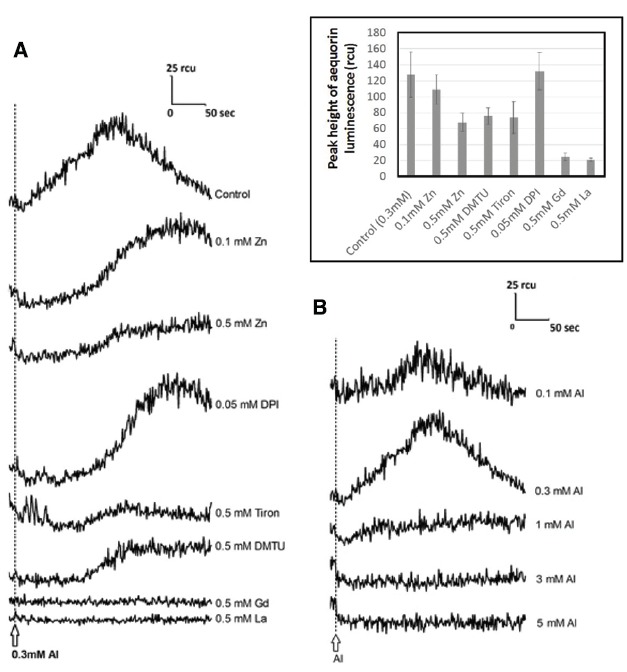
**Monitoring of Al^3+^-responsive [Ca^2+^]_***c***_ with aequorin luminescence in the presence and absence of various inhibitors. (A)** Effect of high- and low-dose Zinc, DPI (NADPH oxidase inhibitor), ROS scavengers (Tiron, DMTU), and trivalent cations (La^3+^ and Gd^3+^) on Al-responsive [Ca^2+^]_*c*_ elevation in tobacco cell suspension culture expressing aequorin. (Inset) Quantitative comparison of the action of inhibitors against the peak height of Al-responsive [Ca^2+^]_*c*_ elevation (*n* = 3; error bars, SD). **(B)** Effect of Al^3+^ concentration on induction of [Ca^2+^]_*c*_ elevation. Typical records of Ca^2+^-responsive aequorin luminescence measured in the presence of different Aluminum concentrations are shown.

To support the hypothesis that Al^3+^-induced distortion in [Ca^2+^]_*c*_ involves the members of ROS derived from the action of NADPH oxidase, and calcium channel opening leading to transient [Ca^2+^]_*c*_ elevation, the effect of DPI (NADPH oxidase inhibitor), ROS scavengers (Tiron, DMTU), and trivalent cations (La^3+^ and Gd^3+^) have been tested in tobacco cells expressing aequorin and compared with the antagonistic action of zinc protecting the cells (Figure 7A and inset).

As expected, Tiron, DMTU, and high concentration of zinc (0.5 mM) effectively lowered the level of Al^3+^-induced [Ca^2+^]_*c*_ elevation. Especially, temporal patterns in which Al^3+^ induces an increase in [Ca^2+^]_*c*_ was shown to be sensitive to both zinc and DPI. In fact, these chemicals significantly retarded the Al^3+^-responsive calcium influx, thus, time required for attaining the peak of Al^3+^-responsive [Ca^2+^]_*c*_ elevation was shown to be longer, suggesting the zinc and DPI might share the common mode of action.

On the other hand, La^3+^ and Gd^3+^ strongly reduce the signal as we observed for high concentration of Al^3+^ (Figure 7A) supporting the view that they can concurrently act inhibiting the Ca^2+^ channel.

**FIGURE 8:**
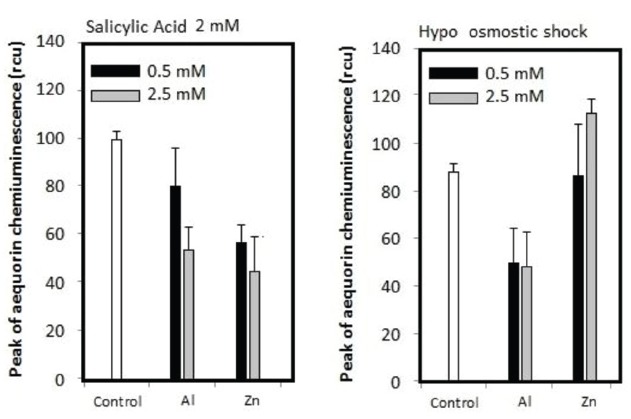
**Effect of Al^3+^ and Zn^2+^ against SA- responsive and hypo-osmotic shock-responsive [Ca^2+^]_***c***_ elevation in tobacco cells.** Error bars, SD; *n* = 3.

**FIGURE 9:**
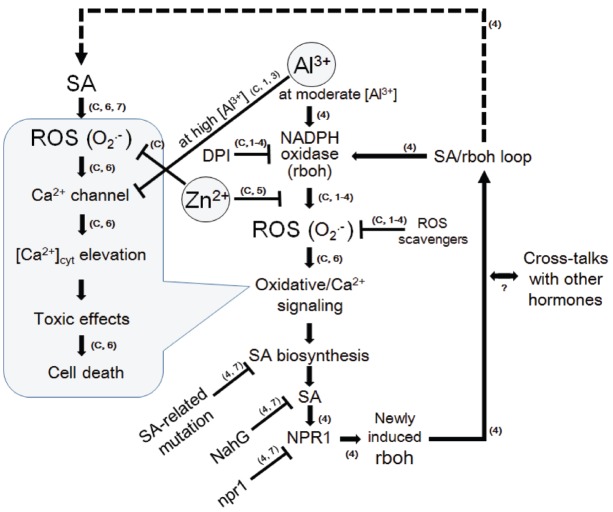
**A model for Al^3+^-induced signaling leading to oxidative cell death and its inhibition by Zn^2+^ in model plant cells.** Knowledge from present experiments using the aequorin-expressing cell lines of tobacco (BY-2) were strengthened by previously reported models. As suggested in the cells of *Arabidopsis thaliana* (Kunihiro et al., 2011), SA signaling pathway is activated in the downstream of ROS such as O_2_^•–^ (and possibly of calcium signaling). The loop of ROS-stimulated the SA signaling and expression of NADPH oxidase which produces further ROS, may eventually lead to development of cell death. Zinc may block the loop by interfering the NADPH oxidase-catalyzed ROS production similarly to the model for lanthanide-induced oxidative burst and cell death in tobacco cells (Kawano et al., 2002). Arrows indicate the stimulation and the T-shaped lines indicate the steps inhibited by agent indicated. The numbers and the symbol “C” in small brackets next to arrows or T-shaped lines denote the source of knowledge, namely; C, data confirmed here; 1, Kawano et al. (2003a; BY-2 cells); 2, Kawano et al. (2004; BY-2 cells); 3, Lin et al. (2005; BY-2 cells); 4, Kunihiro et al. (2011; *Arabidopsis thaliana*); 5, Kawano et al. (2002; BY-2 cells); 6, Kawano et al. (1998; BY-2 cells); 7, Kawano et al. (2013; review on higher plants).

Previously, we have propose a model that Al^3+^ plays dual roles acting for and against the Ca^2+^ influx, by releasing O_2_^•–^ and by inhibiting the Ca^2+^ channel(s), respectively (Kawano et al., 2003a). Al^3+^-dependent distortion in calcium signaling in plant cells can be dissected into two opposing modes of Al^3+^ actions, viz., (i) stimulation of ROS-responsive calcium channels *via* induction of O_2_^•–^ and (ii) inhibition of calcium channels. At low Al^3+^ concentrations, the ROS-responsive Ca^2+^ influx potency is high but the driving force (due to ROS) is not sufficient. At high Al^3+^concentrations, the Ca^2+^ influx-driving force is at sufficient level but the channel’s Ca^2+^ permeability is low. This effect is showed in Figure 7B, where [Ca^2+^]_*c*_ elevation could be manifested only in the range of Al^3+^ concentration in which the two opposing effects eventually compromise (Kawano et al., 2003a). Zn^2+^ hardly blocks the calcium influx in model plant cells unless the event of interest is dependent on the ROS generating events (Figure 8). Therefore, we view here that Zn^2+^ might target only the earlier phase of Al^3+^ action involved in induction of O_2_^•–^ as illustrated in Figure 9.

### The Likely Signaling Paths

In *Arabidopsis thaliana*, Al^3+^-induced prolonged ROS generation requires the expression of *AtrbohD* coding for NADPH oxidase (Kunihiro et al., 2011). This work suggested that biosynthesis and signal transduction pathway for SA is involved in Al^3+^-mediated oxidative burst since the Al^3+^-induced *AtrbohD* expression and cell death were inhibited in the mutant and transgenic cell lines lacking SA biosynthesis, accumulation of SA, and SA-specific signaling components (*sid2*, NahG and npr1, respectively). It has been proposed that loop of SA signal transduction, involving the activity and further induction of NADPH oxidase, forms a signaling circuit enabling an amplification of SA-mediated signaling (Figure 9). This type of oxidative signal amplification was designated as SA/rboh loop (Kunihiro et al., 2011).

By analogy, there would be a similar mechanism in response to Al^3+^ in the cells of tobacco and rice since both the ROS production and cell death were commonly shown to be induced by Al^3+^ in these cells.

Lastly, we propose a likely mode of Zn^2+^ action against Al^3+^-induced cell death. Zn^2+^ may competitively antagonize the action of Al^3+^ by targeting the NADPH oxidase-catalyzed ROS production at upstream of SA signaling mechanism. As a consequence, activation of SA/rboh loop responsible for long-lasting oxidative burst releasing cytotoxic ROS could be prevented (Figure 9).

By assessing the action of Zn^2+^ against SA-induced [Ca^2+^]_*c*_ elevation which is known to be one of the key events in the SA-induced O_2_^•–^-mediated signaling path, involving the activation of Ca^2+^ channel identified as TPC1 channel (Kawano et al., 2013; Lin et al., 2005), we understood that target of antioxidant activity of zinc is not limited to the Al^3+^-induced NADPH oxidase-catalyzed mechanism (Figure 8). It is known that SA-induced rapid O_2_^•–^ is catalyzed by extracellular (cell-wall bound) peroxidase, while SA-induced long-lasting oxidative burst requires the induction of rboh genes coding for NADPH oxidase (Kawano et al., 1998; Yoshioka et al., 2008). In contrast, Zn^2+^ failed to block the Ca^2+^ influx induced by hypo-osmotic shock possibly involving the mechanosensitive-cation channel (Takahashi et al., 1997).

Taken together, target of Zn^2+^ is specifically against the ROS-generating mechanisms (both NADPH oxidase-mediated and peroxidase-mediated) eventually leading to ROS-responsive calcium signaling (Figure 9).

Furthermore, the action of Al^3+^ may form a loop of repeated reaction involving the action of SA which further induces specific type of NADPH oxidase (in case of *Arabidopsis thaliana*, only *AtrbohD* is *Al^3+^-responsive,* Kunihiro et al., 2011).

## Author Contributions

TK designed and supervised the experiments and some key data for plant age and ROS production were obtained by him. CL conducted most tobacco experiments (mostly calcium signaling and cell viability tests), AH and DC performed additional experiments. AH was in charge of rice cell experiments (both ROS detection and calcium signaling). DC and FB contributed on the analysis of data and writing of MS. All authors actively contributed in the discussion.

### Conflict of Interest Statement

The authors declare that the research was conducted in the absence of any commercial or financial relationships that could be construed as a potential conflict of interest.

## Abbreviations

CL: chemiluminescence
CLA: *Cypridina* luciferin analog
DAI: days after inoculation
DPI: diphenylene iodonium
O_2_^•–^: superoxide anion radical
rcu: relative chemiluminescence units
ROS: reactive oxygen species
SA: salicylic acid.
